# Cu-TCPP Metal–Organic Nanosheets Embedded Thin-Film Composite Membranes for Enhanced Cyanide Detection and Removal: A Multifunctional Approach to Water Treatment and Environmental Safety

**DOI:** 10.1021/acsami.4c18944

**Published:** 2025-02-02

**Authors:** Upendar Reddy Gandra, Ravi P. Pandey, L. Palanikumar, Ahamad Irfan, Mazin Magzoub, Youssef Belmabkhout, Shadi W. Hasan, M. Infas Haja Mohideen

**Affiliations:** † Department of Chemistry, Khalifa University of Science and Technology, P.O. Box 127788 Abu Dhabi, United Arab Emirates; ‡ Department of Chemical and Petroleum Engineering, Khalifa University of Science and Technology, P.O. Box 127788 Abu Dhabi, United Arab Emirates; § Center for Membranes and Advanced Water Technology (CMAT), Khalifa University of Science and Technology, P.O. Box 127788 Abu Dhabi, United Arab Emirates; ∥ Biology Program, Division of Science, New York University Abu Dhabi, P.O. Box 129188 Abu Dhabi, United Arab Emirates; ⊥ Center for Catalysis and Separations, Khalifa University of Science and Technology, P.O. Box 127788 Abu Dhabi, United Arab Emirates; # Technology Development Cell (TechCell), Technology Transfer Office (TTO), Mohammed VI Polytechnic University (UM6P), P.O. Box 43150 Ben Guerir, Morocco

**Keywords:** cyanide detection, fluorescence enhancement, thin-film composite membranes, environmental risks, water treatment, filtration, bioimaging agent, excellent lower detection limit (LOD)

## Abstract

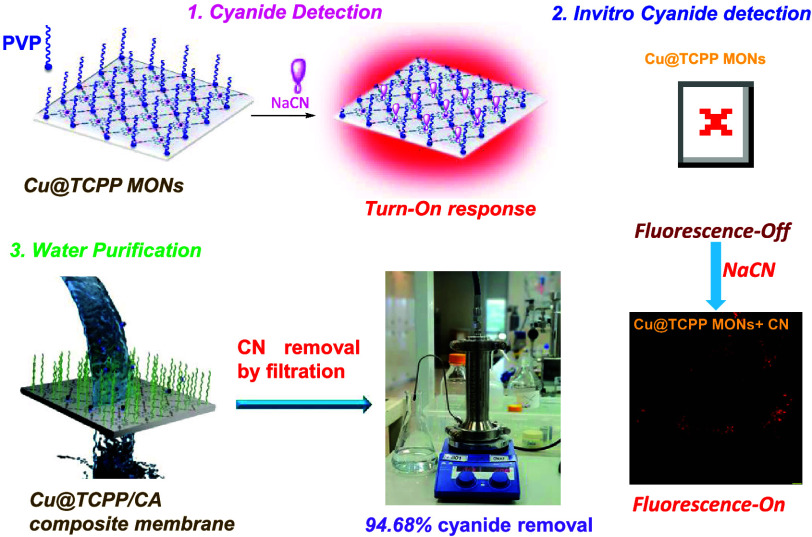

Cyanide is highly toxic, with widespread industrial use posing serious environmental risks. Effective materials for detecting and filtering cyanide from water are urgently needed. This study introduces a novel approach utilizing Cu-TCPP (TCPP = *o*-tetra(4-carboxyphenyl)porphine) metal–organic nanosheets (MONS) embedded in thin-film composite membranes, offering a multifunctional solution for cyanide detection and filtration. Ultrathin Cu-TCPP MONs were synthesized using a surfactant-assisted method featuring highly accessible metal centers that enhance cyanide interaction and detection. The membranes, developed by modifying cellulose acetate (CA) with Cu-TCPP MONs, demonstrated exceptional performance for cyanide removal. The 6% Cu-TCPP/CA membrane exhibited a 2.3-fold increase in pure water permeability and achieved a cyanide removal efficiency of 94.68%, significantly outperforming the pristine 0% Cu-TCPP/CA membrane (Pure Water Permeability (PWP) = 380.83 L m^–2^ h^–1^ bar^–1^; CN^–^ removal = 5.01%). This is the first report describing the detection and removal of CN^–^ in water using the membrane technique in literature. In addition to its removal efficiency, the Cu-TCPP MONs showed remarkable detection capabilities, with a calculated limit of detection of 1.76 × 10^–7^ M, surpassing World Health Organization (WHO) and United States Environmental Protection Agency (EPA) safety standards for cyanide levels in water. Additionally, Cu-TCPP MONs, a bioimaging agent with excellent cell viability, were deployed to detect CN^–^ in MiaPaCa-2 cells, detecting concentrations as low as 0.1 ppm.

## Introduction

Cyanide (CN^–^), a well-known hazardous substance, poses a significant threat to living organisms by disrupting normal respiration in the mitochondrial respiratory chain through binding to cytochrome c.^1,2^ Despite its severe health implications for humans, cyanide continues to be extensively utilized as a reagent across diverse industries such as metallurgy, gold mining, electroplating, plastics manufacturing, etc.^3,4^ Cyanide-contaminated industrial waste has the potential to pollute foodstuffs and water resources, posing a significant threat to human life.^5^ Higher levels of cyanide accumulation can occur through the consumption of specific foods and plants including many food crop species containing sorghum, almonds, flax, bamboo shoot, cherry kernel, sprouting potato, cassava and so on.^2,6−8^ Additionally, CN^–^ serves as a chemical warfare agent.^9^ Recognizing its lethal nature, the widespread industrial application of cyanide, coupled with growing concerns about its potential malicious use, has sparked a recent surge in interest in developing appropriate materials for the selective detection and removal of CN^–^ in water.

Several methodologies have been explored in the literature to design efficient CN^–^ sensors. These include recognition through hydrogen-bonded adduct formation, chemo-dosimetric detection, and utilizing metal-cyanide ion coordination in metal ion-based receptors.^1,10−15^ However, most reported CN^–^ probes, primarily derived from small organic molecules, suffer from several limitations.^4,15−22^ These include poor selectivity, require multistep synthesis, slow response toward cyanide ions, and high detection limits. Additionally, while the design and synthesis of small molecular luminescent sensors for selective CN^–^-detection through chemical modifications hold promise, their reliance on complex organic synthesis methods often presents obstacles. Despite the utilization of various organic and inorganic-based chemosensors and chemo dosimeters to date, the pursuit of a new class of materials capable of providing a platform for the aqueous-phase detection of toxic anions at very low concentrations remains a major focus of interest at present.

In this context, luminescent metal–organic framework Nanosheets (LMONs) have recently surfaced as a distinctive category of two-dimensional (2D) materials characterized by programmable structures, rendering them highly versatile in many applications.^23,24^ Relative to bulk Metal–Organic Frameworks (MOFs), 2D MONs boast larger lateral dimensions and remarkably thin thicknesses, affording them increased accessibility to active sites on their surfaces, thus bestowing them with advantageous sensing capabilities.^25^ Their expansive surface areas, coupled with readily available active sites, diverse surface chemistries, and customizable optoelectronic properties owing to their nanoscale dimensions, have facilitated their integration into various realms, such as light absorption and emission, energy storage mechanisms, and other electronic devices. Moreover, their anisotropic structure and porous nature have positioned MONs as promising candidates for numerous gas separation and water purification processes.^24,26^ Despite these remarkable attributes, the application of LMONs in sensing contexts remains at an early stage of development. While the potential for these nanomaterials in sensing applications is indeed promising, their practical implementation and widespread adoption are still in the initial phases of exploration and refinement. Till now, a very limited number of 2D MON sensors have been reported for various important analytes.^27−32^ Moreover, only a limited number of MOF-based sensors have been reported for the detection of CN^–^.^33−39^ However, some of these sensors suffer from issues related to selectivity and sensitivity, often exhibiting a *turn-off response* upon cyanide binding.^33,37^ Notably, the use of LMONs for detection and adsorption/removal of cyanide from water has not been reported so far.^10^

We are interested in designing and synthesizing highly porous MOF materials derived from various fluorophores with multicentered metal chelates aimed at pollutant detection, bioimaging, and water filtration applications.^40−42^ Specifically, we aimed to integrate the materials into the mixed matrix membranes (MMMs) for water treatment applications.^43,44^ Consequently, employing MONs as an interlayer nanofiller in membrane matrices introduces a novel approach to enhance the separation performance. Moreover, the layer-by-layer assembly of MONs nanosheets allows precise control over interlayer thickness, facilitating exploration into its impact on membrane separation efficiency.^23^ Till now, various adsorbents and photocatalytic materials, such as TiO_2_, ZnO, carbon-based materials, g-C_3_N_4_, and diatomite-based adsorbents, have been utilized for the removal of toxic cyanide ions,^10^ the utilization of MMM for detection and separation studies remains largely unexplored in existing reports. Membrane processes offer several advantages over adsorbent and photocatalytic methods for water treatment.^45^ Membrane technology has extraordinary efficiency in tiny pollutants removal based on size, charge, or chemical structures. They are extremely selective, especially in porous membrane filtration systems, ultrafiltration (UF), nanofiltration (NF), and reverse osmosis (RO), effectively removing particles, bacteria, viruses, and monovalent and divalent salts.^45^

**Figure 1:**
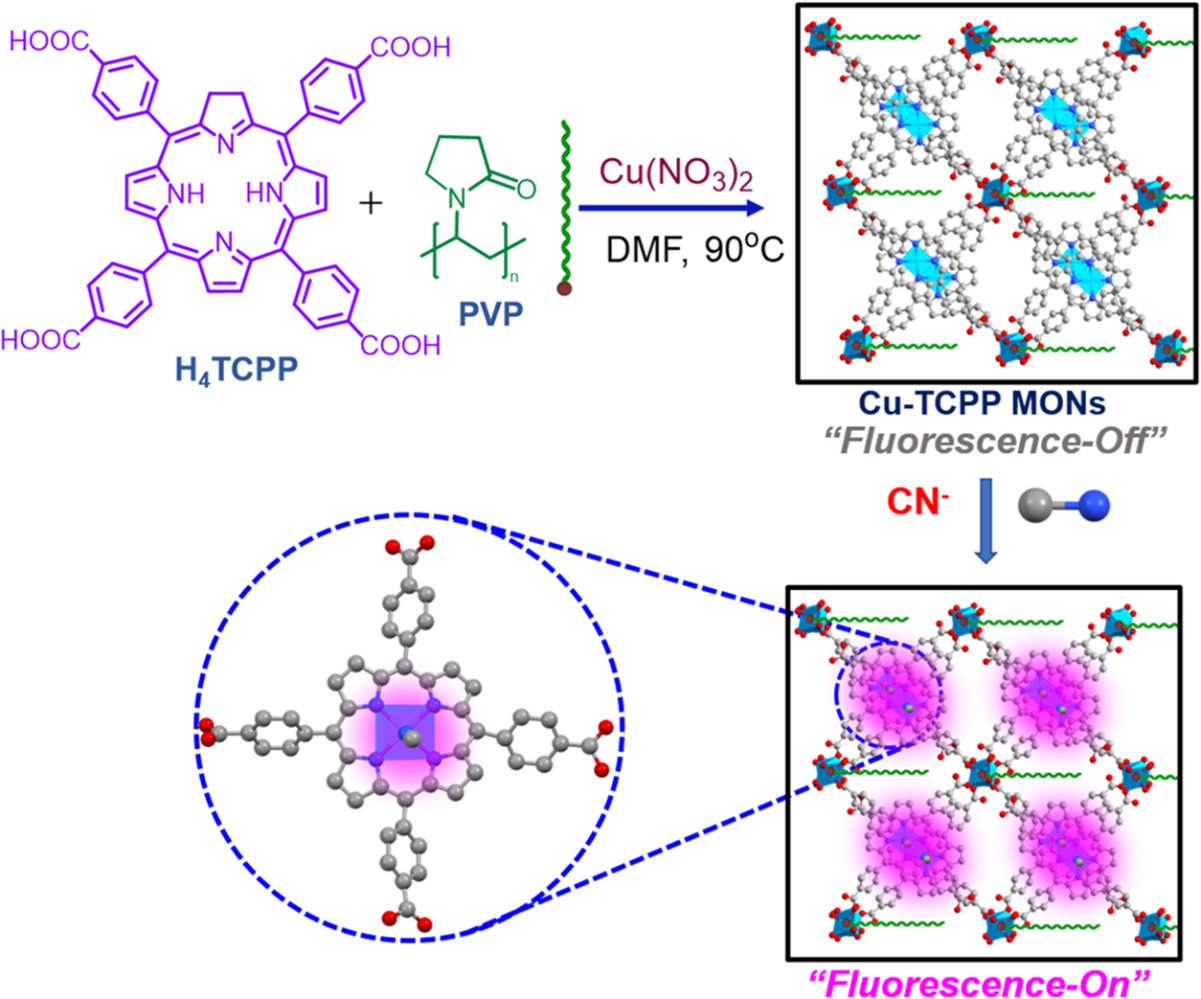
Schematic illustration of the synthesis of Cu-TCPP MONs and sensing mechanism of cyanide.

In this study, we employed a surfactant-assisted synthetic method, utilizing *o*-tetra(4-carboxyphenyl)porphine (TCPP) as the linker, Cu as the metal node, and poly(vinylpyrrolidone) (PVP) as the surfactant, as illustrated in Figure 1.^46^ In Cu-TCPP MONs, TCPP is coordinated with four Cu nodes in a tetradentate fashion that leads to the formation of square pyramidal paddlewheel secondary building units that extend to 2D layers. The surfactant PVP molecules can selectively attach to the surface of MOFs, which plays a key role in the controlled growth of MOF crystals, leading to the anisotropic growth of MOFs and then the formation of ultrathin MOF nanosheets.^23^ The 2D Cu-TCPP MONs provided a highly tunable platform to optimize the sensing performance of the material, in which TCPP acts as a signaling unit before and after cyanide addition where, the Cu center acts as a receptor for cyanide ions. 2D Cu-TCPP MONs showed superior recognition properties for CN^–^ (solvation energy of cyanide in water −339 kJ mol^–1^) over other similar solvation energies of anions like fluoride, acetate, phosphate, etc.^1^ These 2D Cu-TCPP MONs was also used as an imaging reagent for detection of CN^–^ ions in live HeLa cells with [CN^–^] as low as 0.1 ppm. To remove CN^–^ ions from water, we successfully fabricated a series of MMMs by the incorporation of 2D Cu-TCPP MONs into the cellulose acetate (CA) polymer through a nonsolvent-induced phase-separation (NIPS) process.^44^ The novel fabricated Cu-TCPP/CA membranes were assessed in terms of water contact angle (WCA), pure water permeability (PWP), porosity, and their ability to remove CN^–^ from water.

## Experimental Section

### Materials and Instruments

All reagents and solvents were purchased from commercial sources and used without any further purification. The CA polymer (average *M*_n_ ∼ 30,000 by GPC) was purchased from sigma-Aldrich. Fourier transform infrared (FTIR), spectra were recorded on VERTEX 70, Bruker to analyze the functional groups present in the samples. X-ray diffraction measurements were performed to analyze the crystallinity of the samples on Rigaku Smart Lab II with Cu Kα (λ = 1.5405 Å) radiation source operating at 40 kV and 40 mA. Scanning electron microscopy (SEM, JEOL JSM-7610FFEG-SEM) was employed to observe the morphology. A PerkinElmer fluorescence spectrophotometer was used to scan and titrate fluorescence experiments. LAMBDA 1050 UV/vis/NIR Spectrometer was used for ultraviolet–visible (UV–vis) studies. Nexlon 350x PerkinElmer instrument was used for ICP-MS analysis. All measurements were carried out at ambient temperature.

### Synthesis and Characterization

The synthesis of Cu-TCPP MONs was carried out using a modified version of a previously reported procedure.^23^ 100 mg of TCPP and 2.5 g of poly(vinylpyrrolidone) (PVP) were dissolved in 80 mL *N*,*N*-dimethylformamide (DMF) in a beaker under magnetic stirring. Subsequently, 20 mL of Cu(NO_3_)_2_ stock solution (25 mg/mL of Cu(NO_3_)_2_ in DMF) was added. After stirring for 4 min, the reaction mixture was placed in an autoclave and kept at 80 °C in an oven for 24 h. After 24 h, the DMF was removed by centrifugation. The reaction mixture was washed with ethanol and water 3 times. Finally, the brown colored product was collected by centrifugation after washing with acetone. Subsequently, it was left overnight at 100 °C for drying. The resulting product underwent thorough characterization through Powder X-ray diffraction (PXRD), X-ray photoelectron spectroscopy (XPS), UV–vis, fluorescence, IR, and SEM analyses, providing detailed insights into its properties (Figures SI1–S3).

### General Experimental Procedure for UV–Vis and Fluorescence Studies

A stable Cu-TCPP MONs suspension was prepared by taking 3 mg of MON per mL of DMSO and sonicating it for 30 min. Afterward, the suspension was left over for 12 h in an undisturbed state. All of the UV–vis and fluorescence sensing experiments were performed using the suspension prepared above-described manner. This sensing experiment was executed in a quartz cuvette in which we added 3000 μL of water: DMSO and 200 μL of MON suspension. The recording range of the fluorescence emission spectra was 590–800 nm, fixing the excitation wavelength at 580 nm.

### Cell Culture Procedure

Dimethyl sulfoxide (DMSO), Dulbecco’s Modified Eagle Medium (DMEM), Dulbecco’s phosphate buffer saline (PBS), Fetal bovine serum (FBS), 4-(2-hydroxyethyl)-1-piperazineethanesulfonic acid (HEPES), l-glutamine, penicillin/streptomycin antibiotics, sodium pyruvate, trypan blue and trypsin-EDTA solutions were all obtained from Sigma-Aldrich (St. Louis, MO). LysoTracker Green DND-26 and MitoTracker Green were obtained from Thermo Fisher Scientific (Waltham, MA). The CellTiter 96 Aqueous One Solution (MTS) Cell Proliferation Assay Kit was from Promega (Madison, WI). The study utilized Human Pancreatic Cancer cells (MIA PaCa-2) sourced from the American Type Culture Collection (ATCC-CRL-1420). These cells were maintained in DMEM medium, which was supplemented with 10% FBS, 4 mM l-glutamine, 1 mM sodium pyruvate, and 1% penicillin/streptomycin (all acquired from Sigma), and cultured at 37 °C in 5% CO_2_. The viability of the cells was routinely assessed using the Trypan Blue exclusion test on a Bio-Rad TC20 automated cell counter. Upon reaching approximately 95% confluence, the cells were harvested by treating them with 0.25% trypsin-EDTA for subsequent experiments.

### Cancer Cell Uptake

Intracellular imaging was done according to a previously published protocol.^47−49^ Briefly, MIA PaCa-2 cells were seeded at a density of 2 × 10^5^ cells per well in 500 μL of complete medium within 4-chambered 35 mm glass bottom Cellview cell culture dishes (Greiner Bio-One; Monroe, NC). They were then cultured for 24 h at 37 °C in 5% CO_2_. Following this incubation period, the medium was replaced with fresh medium containing Cu-TCPP MONs at a concentration of 5 μM and incubated for an additional 1 h. Subsequently, the media in the chambers were once again replaced with fresh media containing different concentrations of CH_3_CN (0.00, 0.10, 0.15, 0.20, 0.25, 0.30, 0.35, 0.40, 0.45, 0.50, and 1.00 ppm, respectively, prepared in HEPES buffer, pH 7.6) before the cells underwent imaging on a Leica Stellaris 8 Confocal Microscope equipped with a 40× oil immersion objective with DIC capability. Image acquisition was conducted using the LAS X software, and analysis was performed using the Fiji image processing software.^50^

### Co-Localization Analysis

To conduct intracellular imaging for the colocalization of organelles such as mitochondria and lysosomes, MIA PaCa-2 cells were initially seeded at a density of 2 × 10^5^ cells per well in 500 μL of complete medium within 4-chambered 35 mm glass bottom Cellview cell culture dishes (Greiner Bio-One; Monroe, NC). These cells were then cultured for 24 h at 37 °C in 5% CO_2_. After this incubation period, the medium was replaced with fresh medium containing Cu-TCPP MONs at a concentration of 5 μM, and the cells were further incubated for an additional 30 min. Following this, the cells were stained with either LysoTracker or MitoTracker at a concentration of 50 nM for 30 min at 37 °C, following established procedures.^47−49^ Subsequently, the media in the chambers were again replaced with fresh media containing different concentrations of CH_3_CN (0.25 and 0.35 ppm, prepared in HEPES buffer, pH 7.6). Confocal images were then acquired using a Leica Stellaris 8 Confocal Microscope equipped with a 40× oil immersion objective with DIC capability. Image acquisition was conducted using the LAS X software, and subsequent analysis was performed using the Fiji image processing software.

### Cell Viability

To explore the cytotoxic effects of Cu-TCPP MONs, the CellTiter 96 AQueous One Solution (MTS) assay was employed. The assay procedure followed previous protocols.^51^ Initially, MIA PaCa-2 cells were seeded at a density of 5 × 10^3^ cells per well in 100 μL of complete medium in standard 96-well plates. After incubation (37 °C/5% CO_2_) for 24 h, the medium was substituted with fresh medium containing Cu-TCPP MONs at varying concentrations (0.625, 1.25, 2.5, 5.0, 7.5, 10.0, 15.0, and 20.0 μg/mL), and the cells were further incubated for 48 h. Subsequently, the medium was replaced with fresh medium containing 20 μL of MTS reagent, and the plates were incubated for an additional 4 h. The absorbance of the formazan product (λ = 490 nm), resulting from MTS reduction, was then measured using a BioTek Synergy H1MF Multi-Mode Microplate Reader. Control wells were treated solely with the carrier, while wells with medium alone served as blanks. Cell viability was calculated based on the ratio of formazan absorbance in Cu-TCPP MONs-treated cells to that in buffer-treated controls.

### Statistical Analysis

The error bars in this investigation denote the mean ± standard deviation (mean ± SD) of a minimum of three independent replicates (i.e., *n* = 3). Statistical analysis was conducted utilizing GraphPad Prism (version 8.4.2). Significance between the groups was assessed by one-way ANOVA followed by Dunnett’s post hoc test, with *P < 0.05, **P < 0.01, ***P < 0.001 indicating statistical significance.

### Fabrication of Membranes

Cu-TCPP/CA membranes were prepared using the nonsolvent-induced phase-separation (NIPS) method.^44^ Cu-TCPP MONs ranging from 0 to 6 wt % relative to CA were dispersed in *N*-methyl-2-pyrrolidone (NMP) through bath sonication for 1 h in an ultrasonic bath sonicator at room temperature (25 °C). Subsequently, PVP (2 wt %) was slowly introduced into the Cu-TCPP MONs/NMP solution under constant stirring (250 rpm) at 25 °C. Then, CA was gradually added, and the mixture was heated to a temperature between 70 and 75 °C for 2 h. Afterward, the mixture was stirred continuously for an additional 24 h at 25 °C and degassed under a vacuum oven (230 mbar). The resulting dope solution was spread onto a glass plate using a doctor’s blade to achieve an optimized thickness. The glass plate was then submerged in a water coagulation bath containing deionized water at 15 ◦C. Membranes with varying compositions were fabricated and labeled as 0%Cu-TCPP/CA, 2%Cu-TCPP/CA, 4%Cu-TCPP/CA, and 6%Cu-TCPP/CA, where the numerical values represent the weight percentage of Cu-TCPP relative to the CA polymer matrix.

### Instrumental Characterization of Membranes

The membrane water contact angle (WCA) and surface free energy were determined using the KRUSS Drop Shape Analyzer DSA25. The porosity of the developed membranes were determined by a typical gravimetric method using eq (S1) (Section S1, Supporting Information (SI)).^52^ Detailed procedures for the calculation of pure water permeability (PWP), and CN^–^ removal from constructed membranes are provided in the Supporting Information (Section S2). Energy Dispersive X-ray spectroscopy (EDS) was performed on a Phenom SEM desktop instrument. Inductively coupled plasma mass spectrometry (ICP-MS) was performed on (Nexlon 350x PerkinElmer) to detect any leached amount of Cu-TCPP in water during filtration.

## Results and Discussion

**Figure 2:**
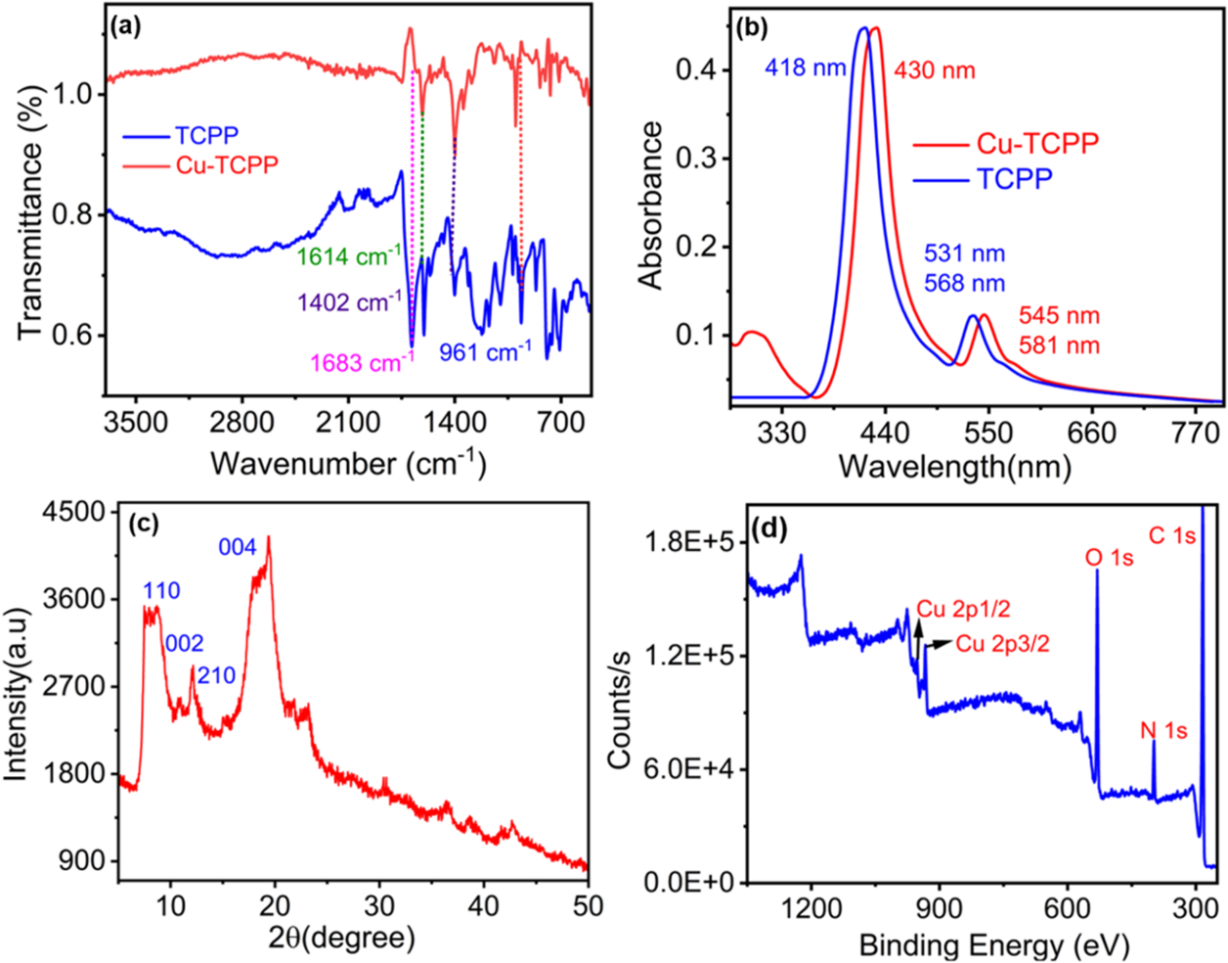
(a) FTIR spectra of H_4_TCPP (blue) and Cu-TCPP MONs (red). (b). Liquid state UV–vis absorption spectra of Cu-TCPP MONs (red) and H_4_TCPP (blue) in aqueous medium. (c) XRD of Cu-TCPP MONs. (d) XPS spectrum for the Cu-TCPP MONs.

The 2D Cu-TCPP MONs obtained were subjected to characterization using powder X-ray diffraction (XRD), X-ray photoelectron spectroscopy (XPS), Infrared (IR) spectroscopy, and UV–vis studies. The IR spectrum of H_4_TCPP showed that the band at 1683 cm^–1^ corresponds to the C=O stretching vibration peak of COOH, while the band at 961 cm^–1^ signifies the N–H stretching vibration peak of pyrrole (Figure 2a).^53^ Upon coordination with Cu^2+^ ions, these characteristic bands disappeared. The FTIR analysis of Cu-TCPP MONs revealed two distinct peaks at 1614 and 1402 cm^–1^, corresponding to O=C–O–Cu bonds, indicating successful coordination bond formation between the COOH of porphyrin rings and Cu^2+^ cations. Additionally, the absence of the peak at 961 cm^–1^ in Cu-TCPP MONs suggests that the N–H in the H_4_TCPP ligand coordinated with Cu ions. These findings were corroborated by UV–vis studies (Figure 2b). The Soret band at 416 nm, characteristic of the porphyrin ring in free H_4_TCPP molecules, exhibited a slight red shift (430 nm) in the Cu-TCPP MONs. Furthermore, while the free H_4_TCPP ligand displayed Q bands in the 531–568 nm range, the Cu-TCPP MONs exhibited two prominent Q bands at 545 and 581 nm, respectively. These results confirm the successful metalation of porphyrin rings by Cu^2+^ ions.

In the XRD spectrum, the broad diffraction peak observed at approximately 18° corresponds to the (004) crystallographic planes of the Cu-TCPP phase, a characteristic feature of 2D nanosheets (Figures 2c and S1). This peak is commonly associated with the layered structure of Cu-TCPP nanosheets and is consistent with the reported diffraction patterns in the literature, further validating the structural assignment. The similarity with previously published data confirms the reliability of our XRD analysis in identifying the Cu-TCPP phase.^23,46,54^ These findings provide strong evidence for the successful synthesis of Cu-TCPP MONs. The peak at 942.74 eV in the XPS spectrum was identified as the shakeup satellite peak associated with Cu^2+^ ions (Figure 2d). Additionally, the presence of a peak at 932.17 eV (Cu 2p_3/2_) and another at 952.9 eV (Cu 2p_1/2_), along with two shakeup satellites within the Cu 2p spectrum, provided clear evidence for the presence of Cu^2+^.^46^ The SEM image clearly visualizes the Cu-TCPP MONs, highlighting their distinctive morphology and confirming their nanosheet-like structure (Figure S3).

### Optical Properties

**Figure 3:**
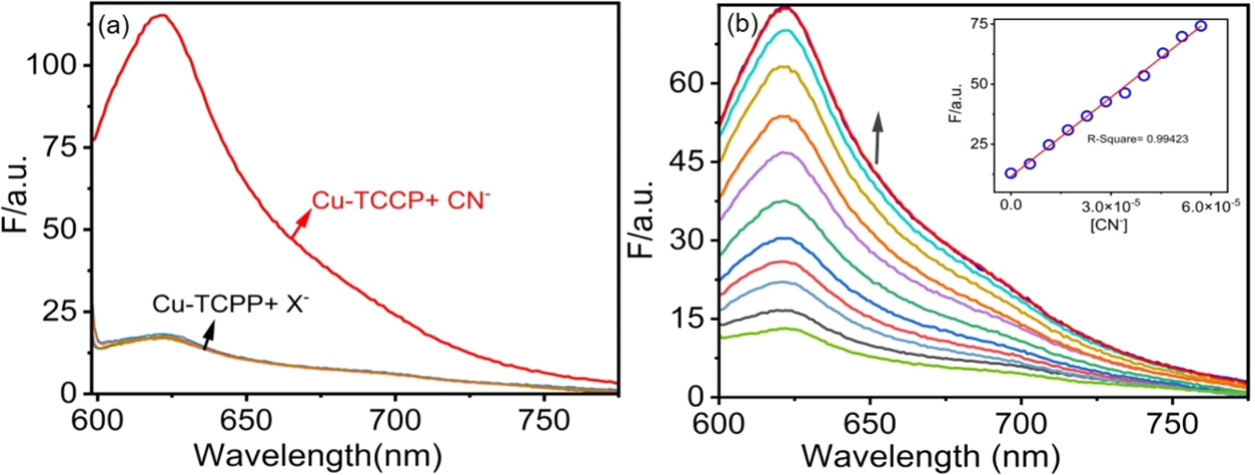
(a) Luminescence spectra (λ_Ext_ = 580 nm) of Cu-TCPP MONs in the absence and presence of various anions (X^–^ = F^–^, Cl^–^, Br^–^, I^–^, CN^–^, CH_3_CO_2_^–^, H_2_PO_4_^–^, HSO_4_^–^, NO_3_^–^, NO_2_^–^, N_3_^–^, ClO_4_^–^ and PhCO_2_^–^); (b) Changes in luminescence spectra for Cu-TCPP MONs in the presence of varying [CN^–^] inset: luminescence titration profile of Cu-TCPP MONs toward varying [CN^–^] (0 to 6.0 × 10^–5^ M) with λ_Ext_ = 580 nm and λ_Emis_ = 622 nm.

The absorption and photoluminescence spectra of Cu-TCPP MONs were recorded in an aqueous medium at room temperature. The intense absorption band maximum at 430 nm is assigned to the characteristic Soret band of the porphyrin ring. Whereas the less intense two absorption shoulders at 545 and 581 nm were assigned to Q bands of the Cu-TCPP porphyrin ring. The typical excitation wavelength for H_4_TCPP falls between 400 to 450 nm, while its emission wavelength typically ranges from 470 to 500 nm. In contrast to H_4_TCPP, Cu-TCPP MONs exhibit two distinct excitation wavelength bands, which are sharply observed at 540 and 580 nm (Figure S2). Upon excitation at 580 nm, Cu-TCPP MONs exhibited a weak^3^ MLCT emission band with a λ_max_ of 622 nm due to the presence of the paramagnetic Cu^2+^ ions (d^9^ system) (Figure 3a).^55^ Luminescence spectra of the Cu-TCPP MONs in the absence and presence of various anionic analytes (X^–^) were recorded to examine the luminescence response of these nanosheets toward different common and physiologically significant anionic analytes (Figure 3a). Among all these anions, only CN^–^ showed a turn-on emission response with a maximum of 622 nm (λ_Ext_ = 580 nm). It is worth mentioning that no detectable change in the electronic spectral pattern of Cu-TCPP MONs was observed either upon the addition of CN^–^ or any other anion studied under identical experimental conditions (Figure S4). Enhancement of the luminescence spectra of Cu-TCPP MONs upon addition of CN^–^ to the solution of Cu-TCPP MONs was due to the strong affinity of CN^–^ toward the Cu(II)-center.

Systematic luminescence titrations were performed for Cu-TCPP MONs with varying [CN^–^] (0 to 6 × 10^–5^ M) (Figure 3b). An increase in [CN^–^] caused a concomitant increase in emission intensity at 622 nm (Figure 3b, inset). The observed emission enhancement with increasing cyanide ion concentration is likely due to the interaction between cyanide ions and the Cu(II) centers in the Cu-TCPP MONs. This interaction can alter the electronic environment, where cyanide ions strongly coordinate with Cu(II), modifying the ligand field and suppressing nonradiative decay pathways, ultimately enhancing the fluorescence. Time-dependent fluorometric titration was also conducted after adding 100 μL of [CN^–^] to the MON suspension in water. At 5 s intervals, the emission spectra were recorded up to 1 min. Within 5 s, the fluorescence of Cu-TCPP MONs became enhanced significantly. Therefore, the response time for Cu-TCPP MONs toward the detection of [CN^–^] was 5 s (Figure S5). To determine the limit of detection (LOD), we used the formula *DL* = *K* × Sb1/*S*. Sb1 is the standard deviation of the blank solution.^56,57^ The *S* value was obtained from the slope of the concentration versus fluorescence intensity plot. The obtained value of LOD was 1.76 × 10^–7^ M (Figure S6). Furthermore, a competitive fluorescence study was performed to investigate the interference of the other anions. The results depicted in (Figure S7) show that the fluorescence intensity increased by CN^–^ was not remarkably affected in the presence of the other anions. Therefore, Cu-TCPP MONs can be an extremely discriminating fluorometric sensor for detecting CN^–^ in waters and conducting real sample analysis. Moreover, the emission spectral pattern of Cu-TCPP MONs remained consistent within the pH range of 6–9 (Figure S8). As a result, the pH range of 6–9 was identified as the optimal pH range, demonstrating the potential of Cu-TCPP MONs for detecting CN^–^ under physiological conditions. The light-induced ROS generation from Cu-TCPP MONs was also confirmed by using SOSG probe (Singlet Oxygen Sensor Green). The emission intensity is at 525 nm, corresponding to SOSG increased upon irradiation of the solution of Cu-TCPP MONs (Figure S12).^58^

### In Vitro Cyanide Detection

**Figure 4:**
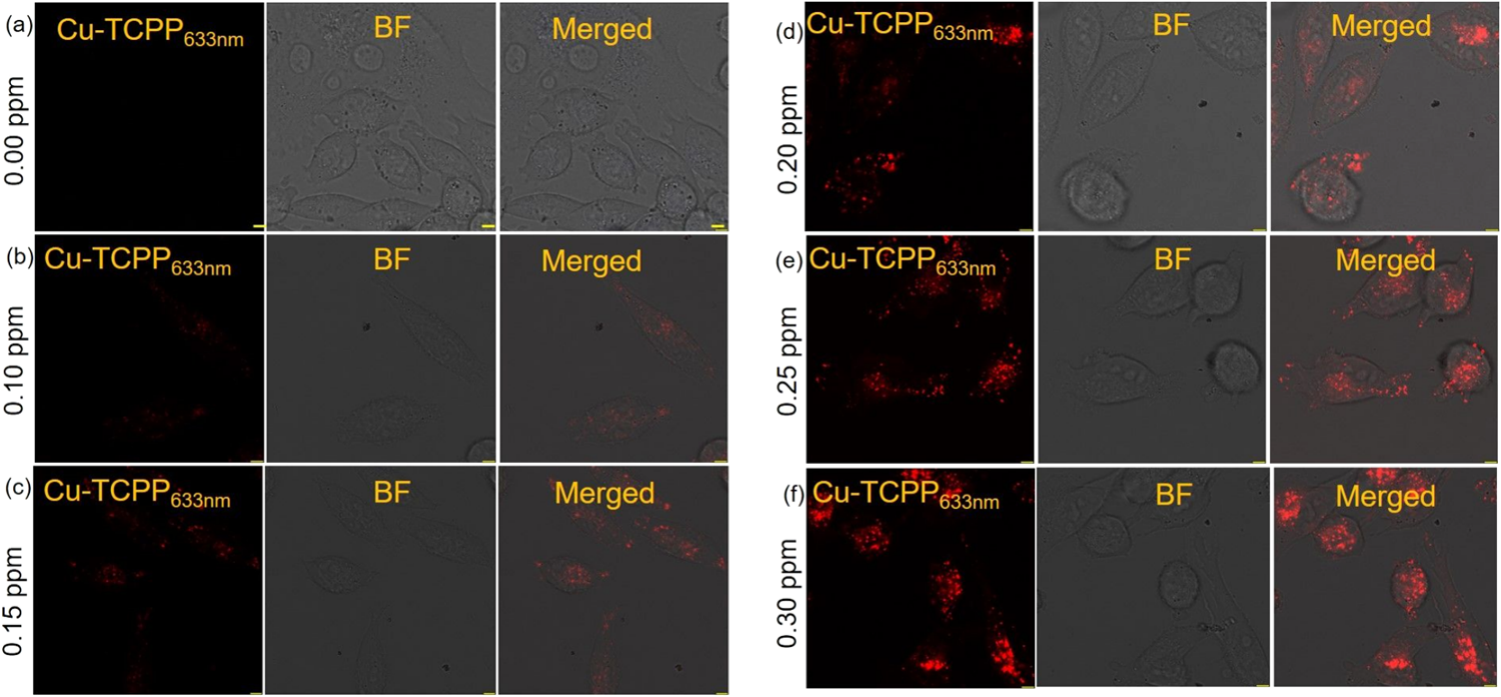
(a–f) Confocal microscope images depicting human pancreatic cancer cells (MiaPaCa-2) following incubation with Cu-TCPP MONs (5 μM) in an aqueous HEPES buffer-CH_3_CN (99.6: 0.4, v/v; pH 7.6) medium, both in the presence and absence of varying concentrations of [CN^–^]. The cells were initially treated with Cu-TCPP MONs for 60 min, after which the media was replaced with fresh media containing different concentrations of Cu-TCPP MONs (ranging from 0.10 to 0.30 ppm [CN^–^], prepared in HEPES buffer, pH 7.6). Subsequently, confocal images were acquired using a Leica Stellaris Microscope, with an emission wavelength set at 633 nm, and were overlaid with the phase contrast images to provide cellular context.

The switch on luminescence response of Cu-TCPP MONs as a function of [CN^–^] offered us the opportunity to use Cu-TCPP MONs as an imaging reagent for the detection of the cellular uptake of CN^–^ in live MiaPaCa-2 cells (epithelial cell line that was derived from tumor tissue of the pancreas) pre-exposed to a buffer solution (pH 7.6) of Cu-TCPP MONs. To examine such a possibility, pretreated live MiaPaCa-2 cells were exposed to various [CN^–^] (0.1 to 1.0 ppm) in aq. HEPES buffer medium (pH 7.6) and were visualized under a laser scanning confocal microscope (Figures 4 and SI9). Initially, Cu-TCPP MONs showed a *turn-off luminescence* response before CN^–^ addition, whereas the micrographs of the loaded cells displayed a distinct increase in the intracellular emission intensity in the red channel (633 ± 10 nm) with an increase in the [CN^–^]. Higher [CN^–^] accounted for the generation of the luminescent Cu-TCPP MONs from the nonluminescent Cu-TCPP MONs, which accounted for the enhanced cellular emission. Further, images shown in Figure 4b confirmed the cell membrane permeability of the Cu-TCPP MONs as well as the possibility of using this reagent for the intracellular distribution of CN^–^ as low as 0.1 ppm, the [CN^–^] set by the U.S. EPA for safe drinking water.^1^ This is significant in the context of the total scenario for the CN^–^ ion detection in an aqueous environment.

**Figure 5:**
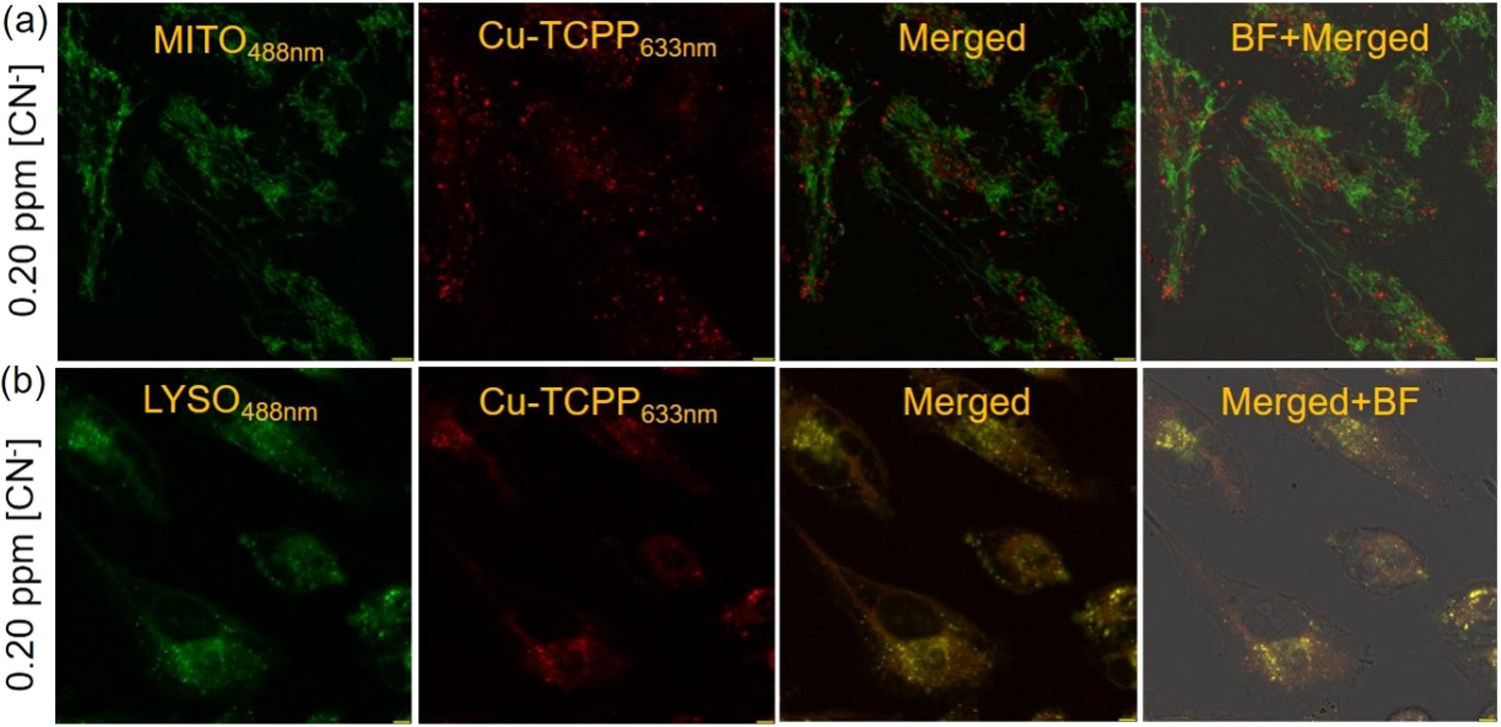
Confocal microscope images showing MiaPaCa-2 cells treated with Cu-TCPP MONs in an aqueous HEPES buffer-CH_3_CN (99.6: 0.4, v/v; pH 7.6) in the presence of [CN^–^] at a concentration of 0.25 (a) or 0.35 (b) ppm and stained with (a) Mito tracker Green and (b) Lysotracker Green to investigate intracellular colocalization.

To determine the potential toxicity effects of the Cu-TCPP MONs, they were tested against MiaPaCa-2 cells by applying a common MTS-based cell viability assay at standard cell culture conditions. MTS assay studies revealed that Cu-TCPP MONs showed insignificant toxicity toward MiaPaCa-2 cells upon incubation for 24 h (Figure S10). Encouraged by the efficacious detection of CN^–^ by Cu-TCPP MONs in Human Pancreatic Cancer cells (MiaPaCa-2), we performed colocalization experiments using organelle-specific fluorescent dyes (Lyso tracker green and Mito tracker green) to identify the location or localization of Cu-TCPP MONs in MiaPaCa-2 cells (Figure 5). Subsequent colocalization analysis employing organelle markers, mitotracker, and lysotracker, revealed a higher rate of colocalization between Cu-TCPP MONs and lysosomes compared to mitochondria within the cancer cells.

### Cyanide Filtration for Water Treatment

**Figure 6:**
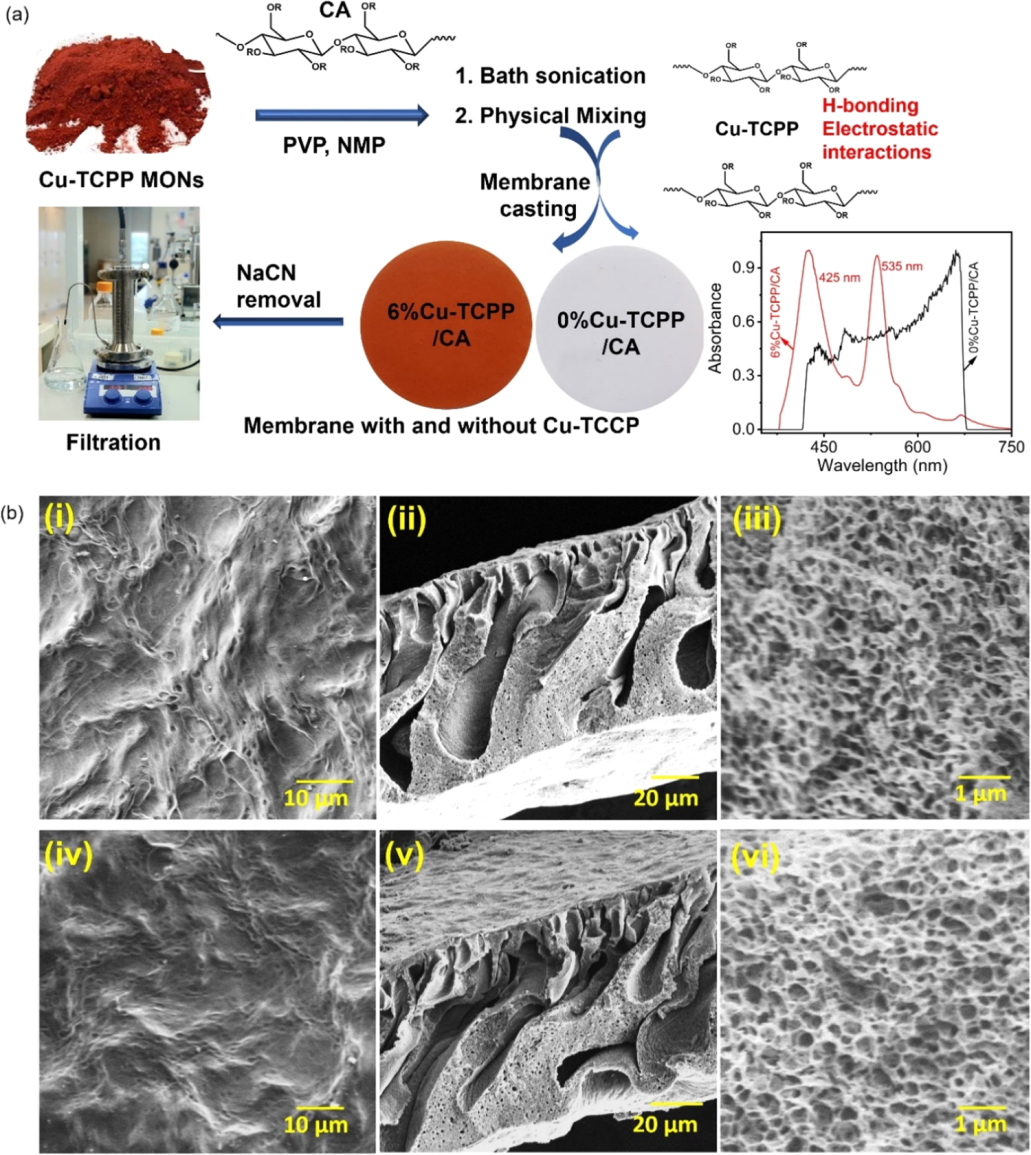
(a) Schematic route for the fabrication of Cu-TCPP/CA composite membranes, structure, possible interactions between Cu-TCPP MONs and CA, and optical images of the 0%Cu-TCPP and 6%Cu-TCPP/CA membranes; (b) solid state UV-spectra of membranes before (blue line) and after (red line) Cu-TCPP casting of (c) SEM images: (i) surface of CA, (ii) cross-section of CA, (iii) high-resolution image of CA cross-section, (iv) surface of 6%Cu-TCPP/CA, (v) cross-section of 6%Cu-TCPP/CA, (vi) high-resolution image of 6%Cu-TCPP/CA cross-section.

The Cu-TCPP/CA membranes were fabricated by NIPS using dope solutions of Cu-TCPP MONs with CA. Schematic membrane synthetic route, membrane chemical structure, possible interactions between Cu-TCPP and CA, optical image of membranes, solid-state UV spectra of 0% Cu-TCPP/CA and 6% Cu-TCPP/CA membranes, and filtration apparatus are shown in Figure 6b.

**Figure 7:**
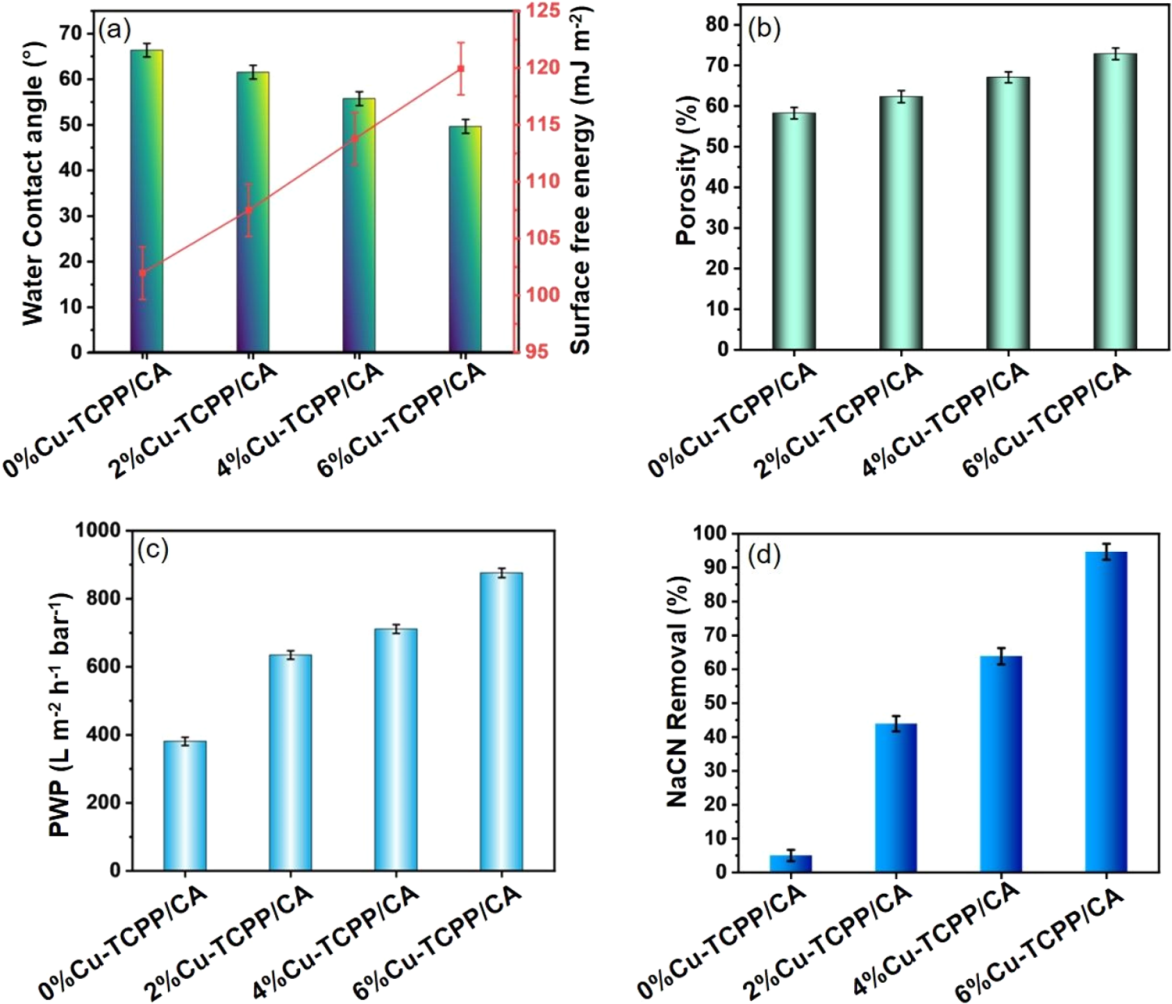
(a) Water contact angle and surface free energy of Cu-TCPP/CA composite membranes; (b) porosity of fabricated membranes; (c) pure water permeability of membranes; and (d) NaCN removal of developed membranes.

It is expected that the Cu-TCPP MONs will stabilize in the CA polymer matrix by forming a strong H-bond between the oxygen-containing groups of Cu-TCPP and CA. The optical images of the 0%Cu-TCPP/CA and 6%Cu-TCPP/CA display white and reddish-brown color, respectively. The presence of Cu-TCPP MON in the 6%Cu-TCPP/CA membrane was confirmed by solid-state UV–vis spectra (Figure 6b). The solid-state UV–vis of 6%Cu-TCPP/CA showed two characteristic bands at ∼425 and ∼535 nm attributed to the Soret and Q bands of Cu-TCPP MONs, this result is further validated with UV–vis solution spectra (Figure 2b). The microstructure of the fabricated 0% Cu-TCPP/CA and 6%Cu-TCPP/CA composite membranes were investigated by SEM and results are shown in Figure 6c. Both membranes showed asymmetric porous structure and were free from any cracks. Cross-sectional images of 0%Cu-TCPP/CA and 6%Cu-TCPP/CA membranes exhibited asymmetric finger-like spongy structures (Figure 6(c(ii,v))). We performed WCA of the fabricated membranes, including 0%Cu-TCPP/CA, 2%Cu-TCPP/CA, 4%Cu-TCPP/CA, and 6%Cu-TCPP/CA (Figure 7a). The WCA of the prepared membranes was decreased with the incorporation of Cu-TCPP into the CA membrane, and 6%Cu-TCPP/CA membrane showed the lowest WCA (49.65°) among all developed membranes. These lower WCA values indicate a significant enrichment in hydrophilicity of 6%Cu-TCPP/CA membrane by incorporating Cu-TCPP. Similarly, membrane surface wettability was evaluated by determining the surface free energy of the membrane. A higher value of surface free energy indicates a facile surface for water molecules, which helps in water permeability. The 6%Cu-TCPP/CA membrane exhibited an enhanced surface free energy of 119.93 mJ m^–2^, in comparison to the pristine 0%Cu-TCPP/CA membrane (101.97 mJ m^–2^). This result showed great affinity of the 6%Cu-TCPP/CA membrane toward water molecules. The porosity of the fabricated Cu-TCPP/CA membranes indicates key properties that directly impact the water permeability and NaCN removal performance Figure 7b. The Porosity of fabricated Cu-TCPP/CA membranes was increased with the addition of Cu-TCPP MON into the CA membrane. The 6%Cu-TCPP/CA membrane revealed the highest porosity (72.85%) among all the fabricated Cu-TCPP/CA membranes. The enhanced porosity of 6%Cu-TCPP/CA membrane can be credited to the increased wettability induced by Cu-TCPP, leading to quicker solvent exchange rates during membrane fabrication by the NIPS method. The PWP of the fabricated Cu-TCPP/CA composite membranes was potentially enhanced by adding Cu-TCPP MON into CA (Figure 7c). The 6%Cu-TCPP/CA membrane presented 2.3 times higher in PWP (875.91 L m^–2^ h^–1^ bar^–1^) than the 0%Cu-TCPP/CA membrane (PWP = 380.83 L m^–2^ h^–1^ bar^–1^). This inclination in PWP can be credited to the construction of additional water pathways by the Cu-TCPP MON. Additionally, the CN^–^ removal rate (%) of the fabricated membranes increased moving from 0%Cu-TCPP/CA membrane to 6%Cu-TCPP/CA membrane (Figure 7d). The 0%Cu-TCPP/CA membrane showed a very low removal of CN^–^ (5.01%), maybe due to its bigger mean pore size (∼74 nm) as compared to the hydrated radius of the CN^–^ (∼17.8 × 10^–2^ nm). However, 6% incorporation of Cu-TCPP into CA significantly enhanced the removal of the CN^–^ (94.68%) for 6%Cu-TCPP/CA membrane. This can be ascribed to the strong attraction ability of Cu-TCPP MOF toward CN^–^, leading to an increased removal rate. Fluorescence experiments were conducted on the filtered water from Cu-TCPP/CA membranes to demonstrate the impact of Cu-TCPP MONs during CN^–^ filtration (Figure S11). In this experiment, 50 ppm of NaCN spiked to water passed through the Cu-TCPP/CA membranes. No change in fluorescence intensity was observed in water samples collected from membranes with a higher percentage of incorporated Cu-TCPP MONs. To assess the stability of Cu(II) in the 6%Cu-TCPP/CA composite membrane during cyanide filtration, PXRD, EDAX, and ICP-MS analyses were conducted (Figures S13a and S14). PXRD and EDAX analyses revealed no significant changes in the structural or elemental composition of the materials, likely attributed to the low loading of Cu-TCPP. ICP-MS further confirmed the absence of Cu leaching after 8 h of cyanide filtration, demonstrating the robust integration of Cu(II) within the CA matrix. Additionally, solid-state UV–vis spectroscopy indicated no change in the electronic properties of the 6%Cu-TCPP/CA membrane before and after CN^–^ exposure (Figure S13b). The 6%Cu@TCPP/CA membrane reusability test retained about 76.94% flux recovery ratio (FRR) after the 3th cycle. The stepwise drop in FRR from first cycle to third cycle showed a fast decline in the first step (∼12.68%) followed by a slow decline for second (7.79%) and third cycles (4.43%), indicating membrane reusability (Figure S15). This significant result underscores the advantageous luminescence properties of the membranes. Moreover, the effectiveness of removing cyanide from water using this technique has previously been unreported in the literature.

To date, adsorption and photocatalysis studies have been documented in the literature for the removal of cyanide from water, as shown in Table 1. However, these studies require a large time scale, high basic pH conditions, dependence on UV light sources for catalytic degradation, and the necessity for additional chemical components. Membrane filtration presents several significant advantages over adsorption studies, particularly in terms of separation efficiency, scalability, energy efficiency, and the capability for continuous operation.^59^ Unlike adsorption, which is typically a batch process, membrane filtration can be operated continuously, enhancing efficiency and throughput. Moreover, membrane processes generally do not require the addition of chemicals, thereby reducing the risk of chemical contamination and simplifying waste management.^59^ The stability of Cu-TCPP within the membrane was assessed by leaching experiments. The leaching experiment was conducted by placing the best 6%Cu-TCPP/CA membrane in a dead-end filtration setup (HP4370 Sterlitech, Co) with an effective membrane area of about 1.25 × 10^–3^ m^2^ and filter deionized water for 24 h. The permeated water was analyzed using ICP-MS analyzer to detect any leached amount of Cu-TCPP in water during filtration. Remarkably, no significant amount of Cu element was detected, indicating that the Cu-TCPP nanomaterial is stable with the CA membrane matrix.

**Table 1:** Comparison between Previous Work and This Work for the Treatment of CN^–^

sr. no	nanomaterial/membrane	removal method	conditions	removal (%)/adsorption (mg/g)	refs
1	TiO_2_ P25	photocatalytic oxidation	[adsorbent]TiO_2_ (0.05 g/L); KCN (30 ppm)	72.32%	(60)
2	corncob biochar	adsorption	CN^–^ (30 ppm)	2.57 mg/g	(61)
3	ZnO/Fe_2_O_3_/activated carbon (AC)	adsorption	[CN]^−^ = 100 mg/L, pH = 10, time = 24 h, [adsorbent] = 1.5 g/L	82.5%	(62)
5	granular activated carbon	adsorption	pH 13, [Fe(CN)_6_]^3–^ (100 ppm)	76.81%	(63)
6	K_2_S_2_O_8_ 2.0 wt %,	oxidation	temperature = 60 °C, and time = 60 min, pH = 10	62.18%%	(64)
7	TiO_2_–Pd-HAP-FeTCPP/UV–vis	adsorption	[CN^–^] = 100 ppm, pH = 11, Pd–TiO_2_–HAP = 450 mg, Fe-TCPP = 150 mg, time = 90 min	90%	(65)
8	TiO_2_/Fe_2_O_3_/zeolite/UV/H_2_O_2_	adsorption	[CN]^−^ = 200 ppm, pH = 10, time = 160 min, [catalyst] = 1.4 g/L, [H_2_O_2_] = 400 ppm	89%	(66)
9	iron-zeolite	adsorption	[CN]^−^ = 100 ppm, pH = 10.5, time = 12 h, [adsorbent] = 10 g/L	90%	(67)
10	GO/TiO2/ZSM-5-X	photocatalytic oxidation	1.0 h of preadsorption and 3.0 h of GTZ-60 photocatalysis	92.91%	(68)
11	6%Cu-TCPP/CA	membrane filtration	50 ppm	94.68%	this study

These benefits make membrane filtration an optimal choice for numerous industrial and environmental applications that demand precise and efficient separation. In addition, this study marks the first documentation of Cu-TCPP Metal–Organic Nanosheets (MONs) being utilized for fluorometric cyanide sensing, in vitro cyanide detection, and water purification applications. The innovative application of Cu-TCPP MONs underscores the potential of advanced membrane materials to significantly improve the detection and removal of cyanide, paving the way for future research and practical applications in water treatment technologies. These benefits make it a preferred choice for many industrial and environmental applications where precise and efficient separation is crucial.

## Conclusions

In conclusion, the Cu-TCPP MONs exhibit remarkable selectivity in detecting CN^–^ ions over other anions with similar solvation energies. The observed fluorescence enhancement specifically toward CN^–^ underscores the pivotal role of accessible metal centers on the MON surfaces in enhancing detection capabilities. The calculated limit of detection (LOD) for CN^–^ stands at 1.76 × 10^–7^ M, surpassing safety thresholds set by regulatory bodies like the World Health Organization (WHO) and the United States Environmental Protection Agency (EPA). Additionally, the nanosheets can detect CN^–^ ions in live cells at very low concentrations (0.1 ppm). The integration of Cu-TCPP MONs into thin-film composite (TFC) membranes termed (Cu-TCPP/CA) for water treatment applications is successful, which is not reported in the literature. These membranes exhibit promising properties such as water contact angle (WCA), pure water permeability (PWP), and porosity. The development of Cu-TCPP MONs embedded in thin-film composite membranes enhances detection sensitivity and offers efficient cyanide removal up to 94.68%, making this approach a pioneering solution for addressing water contamination challenges.

## Supporting Information Available

Detailed characterization and analyses of Cu-TCPP MONs: simulated XRD patterns; excitation spectra; SEM images; UV–vis spectral responses to anions, and time-dependent fluorescence for CN detection. It also covers detection limit calculations; interference and pH-dependent studies; confocal imaging of MiaPaCa-2 cells with varying [CN^–^]; cell viability analysis, porosity and pore size calculations, PWP and CN^–^ removal, fluorescence in filtered water; singlet oxygen generation, and PXRD/UV–vis data for Cu-TCPP/CA before and after CN^–^ loading; along with recyclability studies (PDF)
