# Supplementary material for: Cu-TCPP Metal–Organic Nanosheets Embedded Thin-Film Composite Membranes for Enhanced Cyanide Detection and Removal: A Multifunctional Approach to Water Treatment and Environmental Safety
Source: ACS Appl Mater Interfaces. 2025 Feb 2;17(6):9563–74. doi: 10.1021/acsami.4c18944 (PMC11826502; doi:10.1021/acsami.4c18944)
Supplement: Supplementary file 1 — am4c18944_si_001.pdf [file am4c18944_si_001.pdf]

Supporting Information

Cu-TCPP Metal-Organic Nanosheets Embedded Thin-Film Composite Membranes for Enhanced Cyanide Detection and Removal: A Multifunctional Approach to Water Treatment and Environmental Safety

Upendar Reddy Gandra[a], Ravi P. Pandey[b,c], Loganathan Palanikumar[d], Ahamad Irfan[a,e], Mazin Magzoub[d], Youssef Belmabkhout[f], Shadi W. Hasan[b,c]*, M. Infas Haja Mohideen [a,e]*

[a]*Department of Chemistry, Khalifa University of Science and Technology, P.O. Box 127788, Abu Dhabi, United Arab Emirates; E-mail: mohamed.mohideen@ku.ac.ae*

[b]*Department of Chemical and Petroleum Engineering, Khalifa University of Science and Technology, PO Box 127788, Abu Dhabi, United Arab Emirates. E-mail address: shadi.hasan@ku.ac.ae*

[c]*Center for Membranes and Advanced Water Technology (CMAT), Khalifa University of Science and Technology, PO Box 127788, Abu Dhabi, United Arab Emirates*

[d]*Biology Program, Division of Science, New York University Abu Dhabi, Abu Dhabi, United Arab Emirates, PO Box 129188*

[e]*Center for Catalysis and Separations, Khalifa University of Science and Technology, Abu Dhabi P.O. Box 127788, United Arab Emirates.*

[f]*Technology development Cell (TechCell), Technology Transfer Office (TTO), Mohammed VI Polytechnic University (UM6P), Ben Guerir, Morocco, PO Box 43150.*

.

Table of contents	Page no
Simulated X-ray diffraction patterns of Cu-TCPP MONs & Excitation spectra of Cu-TCPP MONs	S3
SEM images of Cu-TCPP nano sheets & UV-Vis spectral response of Cu-TCPP MONs towards various anions	S4
Time-dependent fluorescence response of Cu-TCPP MONs in the presence of CN^-	S5
Calculation of detection limit	S5-S6
Interference study in the presence of various anions	S7
Change in fluorescence of Cu-TCPP MONs as a function of the solution pH	S7
Confocal microscope images depicting Human Pancreatic Cancer Cells (MiaPaCa-2) following incubation with Cu-TCPP MONs with varying concentrations of [CN^-] (0.35 to 1.0 ppm)	S8
Cell viability analysis of Cu-TCPP MONs in Human Pancreatic Cancer cells (MiaPaCa-2)	S9
The Porosity calculation:	S9
Pure water permeability (PWP) and CN^- removal	S10
Fluorescence response of Cu-TCPP MONs towards filtered water from Cu-TCPP/CA membranes	S11
Singlet oxygen generation ability of Cu-TCPP MONs	S11
PXRD and solid-state UV-Vis spectroscopy of 6% Cu-TCPP/CA before and after CN^- loading	S12
EDAX analysis of 6% Cu-TCPP/CA before and after CN-loading	S13
Recyclability	S14
Pore Size calculation:	S14

Comparison of simulated X-ray diffraction patterns of Cu-TCPP Bulk with Cu-TCPP MOns

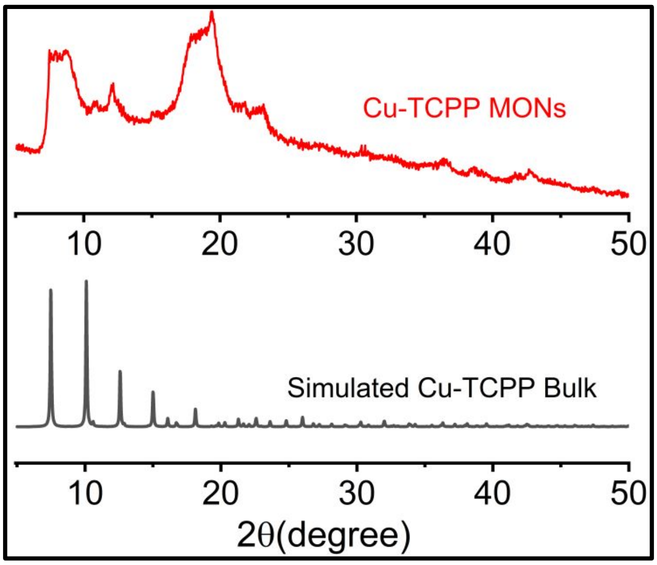

Figure S1. Simulated X-ray diffraction pattern of Cu-TCPP Bulk and PXRD of Cu-TCPP MONs.

Excitation spectra of Cu-TCPP MONs

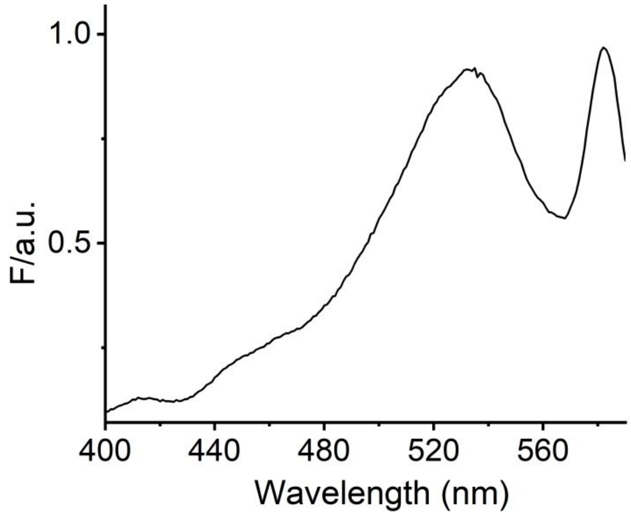

Figure S2. Normalized excitation spectra of Cu-TCPP MONs emission monitored at 622 nm in water.

SEM images of Cu-TCPP nano sheets

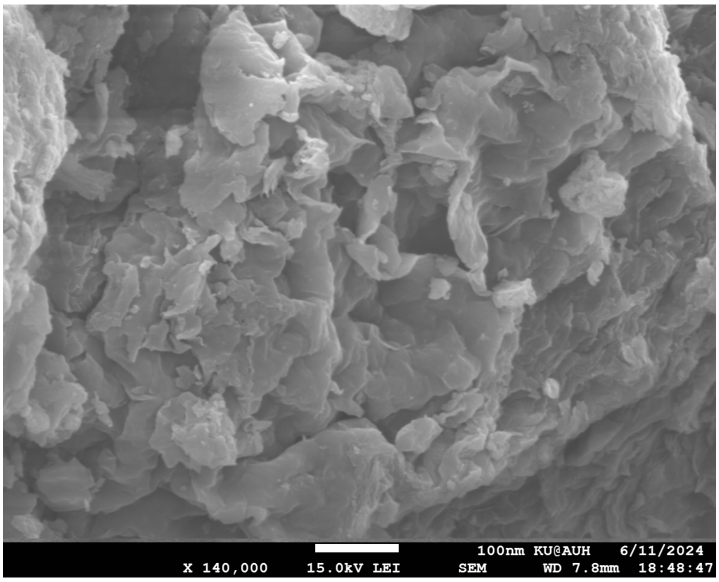

Figure S3. SEM images of Cu-TCPP nanosheets.

Uv-Vis spectral response of Cu-TCPP nanosheets towards various anions

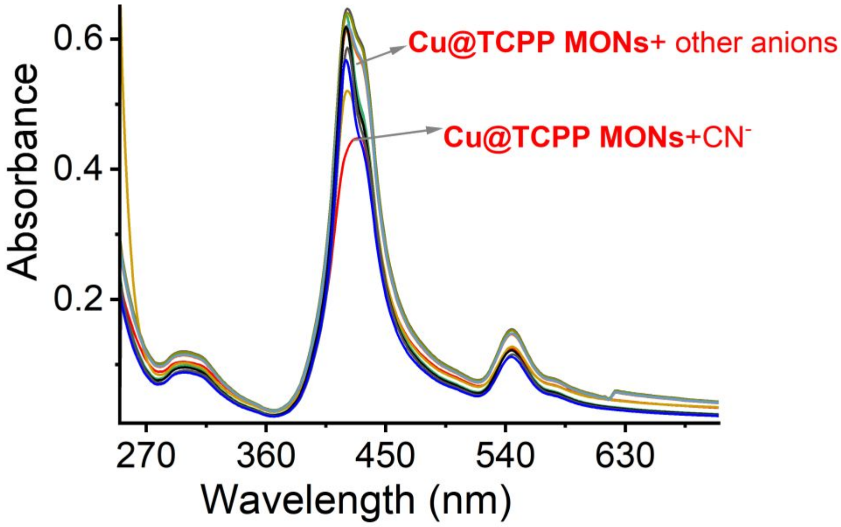

Figure S4. UV-Vis spectral response of Cu-TCPP MONs in the absence and presence of various anions (X^- = F^-, Cl^-, Br^-, I^-, CN^-, $CH_3CO_2^-$, $H_2PO_4^-$, HSO_4^-, NO_3^-, NO_2^-, N_3^-, ClO_4^- and $PhCO_2^-$) in Water.

Time-dependent fluorescence response of Cu-TCPP MONs in the presence of CN^-

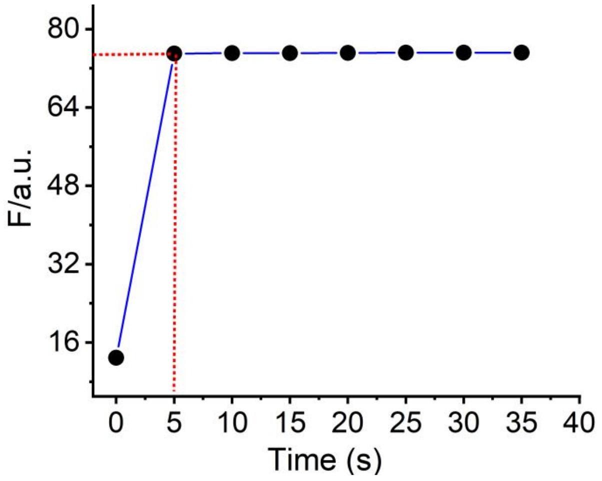

Figure S5. Time-dependent fluorescence spectra (λ_{Ext} = 580 nm; Emission monitored at 622 nm) of Cu-TCPP MONs upon addition of an aqueous solution CN^- (6.0 x10^{-5} M.) in water.

Calculation of detection limit:

The detection limit was calculated based on the fluorescence titration. To determine the S/N ratio, the emission intensity of Cu-TCPP MONs without CN^- was measured 8 times and the standard. The deviation of blank measurements was determined. The detection limit (DL) of Cu-TCPP MONs for CN^- was determined from the following equation:

DL = K * Sb1/S

Where K = 2 or 3 (we took 2 in this case);

Sb1 is the standard deviation of the blank solution; S is the slope of the calibration curve.

From the graph we get slope = 1.097×10^7, and Sb1 value is 0.9624

Thus, using the formula, we get the Detection Limit = 1.76×10^{-7} M.

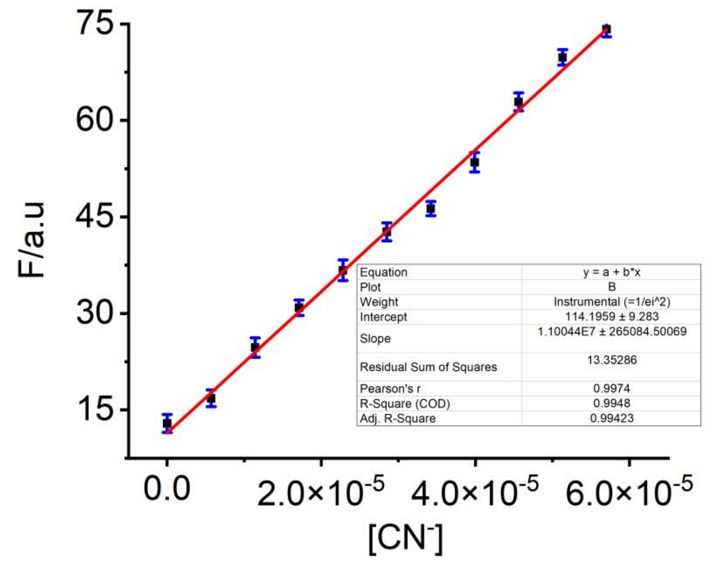

Figure S6. Fluorescence intensity of Cu-TCPP MONs upon addition of CN^- (0- 0.6 μM) in THF: Water (3:1, v/v) using λ_{Ext} = 580 nm.

Interference study in the presence of various anions:

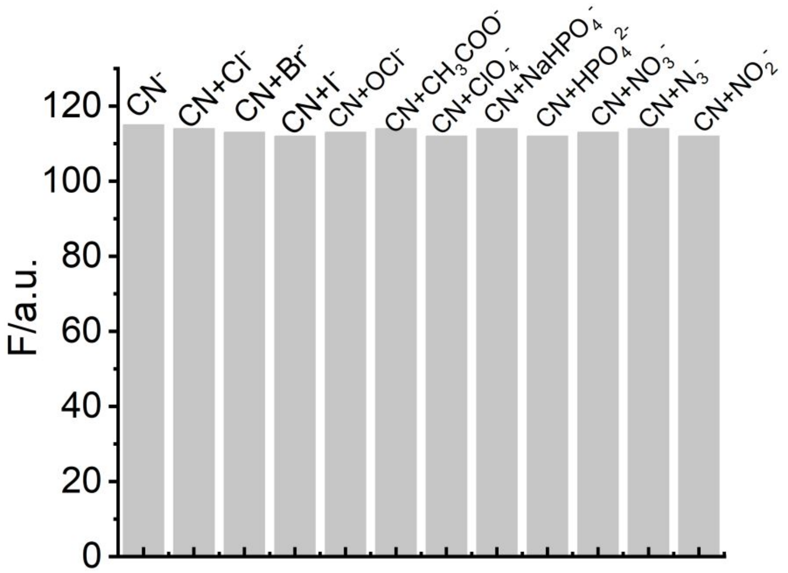

Figure S7. Interference study of Cu-TCPP MONs with CN^- in the presence of different anions (10-mole equiv.) in water using λ_{Ext} = 580 nm.

Change in fluorescence of Cu-TCPP MONs as a function of the solution pH

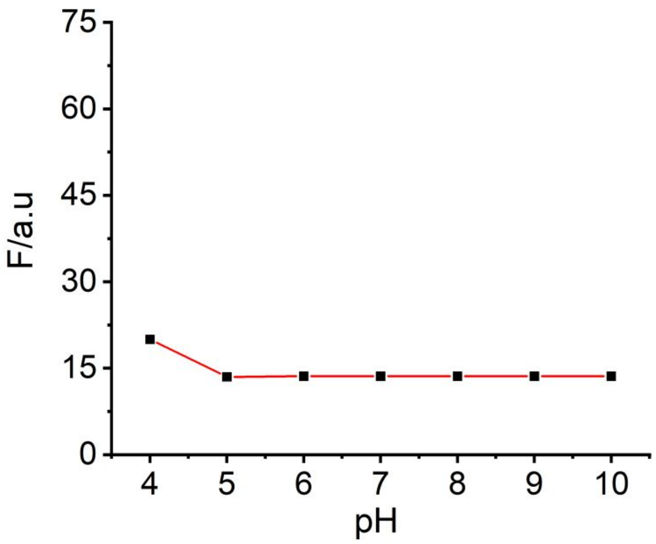

Figure S8. The fluorescence response of Cu-TCPP MONs is a function of pH in a Universal buffer, and pH is adjusted by using aqueous solutions of 1 M HCl or 1 M NaOH.

Confocal microscope images depicting Human Pancreatic Cancer Cells (MiaPaCa-2) following incubation with Cu-TCPP MONs with varying concentrations of [CN⁻] (0.35 to 1.0 ppm)

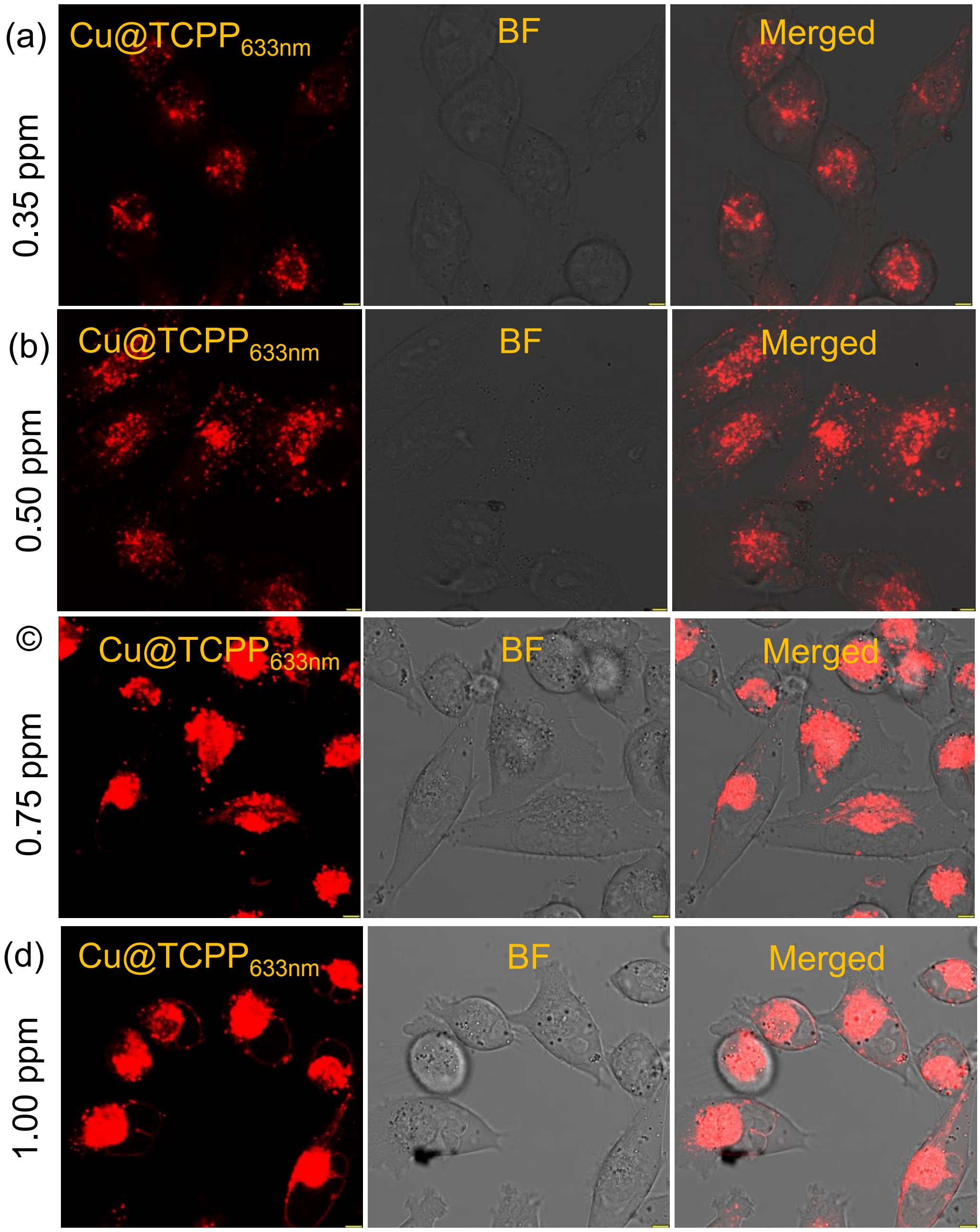

Figure S9. (a-d) Confocal microscope images showing human pancreatic cancer cells (MiaPaCa-2) following incubation with Cu-TCPP MONs (5 µM) in an aqueous HEPES buffer–CH_3CN (99.6: 0.4, v/v; pH 7.6) medium, in the presence of varying concentrations of NaCN. The cells were initially treated with Cu-TCPP MONs for 60 minutes, after which the media was replaced with fresh media containing different concentrations of NaCN (ranging from 0.35 to 1.00 ppm, prepared in HEPES buffer, pH 7.6). Subsequently, confocal images were acquired using a Leica Stellaris Microscope, with emission wavelength set at 633 nm, and were overlaid with phase contrast images to provide cellular context.

Cell viability analysis of Cu-TCPP nanosheets in Human Pancreatic Cancer cells (MiaPaCa-2)

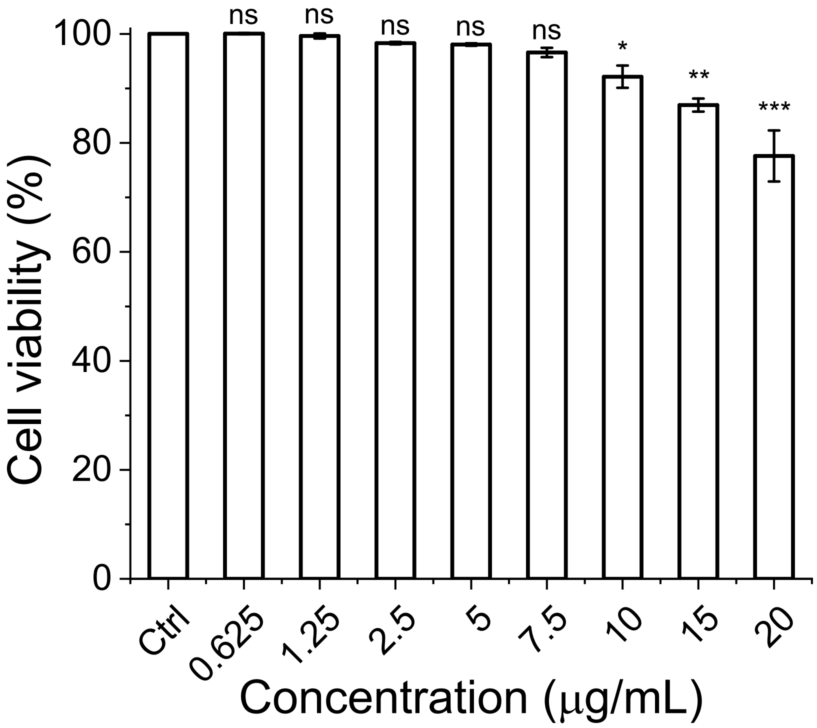

Figure S10. Cell viability analysis of MiaPaCa-2 cells following treatment with varying concentrations of Cu-TCPP MONs (0.625, 1,25, 2.5, 5.0, 7.5, 10.0, 15.0 and 20.0 μg/mL). Cu-TCPP MONs were dispersed in an aqueous HEPES buffer with CH_3CN (99.6: 0.4, v/v, respectively; pH 7.6) before cell treatment. After 24 hours of incubation, cell viability was assessed using the MTS assay. Data are presented in mean ± SD. Statistical analysis was performed using one-way ANOVA followed by Dunnett's post hoc test. **P* < 0.05, ***P* < 0.01, ****P* < 0.001, for comparisons with controls.

The Porosity calculation:

Section S1.

The porosity of the membranes was measured using **Eq. (S1)**:[1]

$$\text{Porosity (\%)} = \frac{(W_{wet} - W_{dry})}{(A\ d\ \rho_m)} \times 100 \qquad \text{(S1)}$$

where, W_{wet} and W_{dry} are the weights of wet membrane after immersing in water for 24 h, followed by removing water from membrane surface and the weight of dry membrane after keeping under vacuum at 60 °C for 48 h to remove all the water content. A, d, and ρ_m are the membrane area (cm^2), membrane thickness (cm), and water density (1.0 g cm^{-3}); respectively.

Section S2. Pure water permeability (PWP) and CN^- removal

The PWP and CN^- removal of the fabricated membranes was assessed using a dead-end filtration setup (HP4370 Sterlitech, Co, USA) with an effective membrane area of about 1.25 × 10^{-3} m^2. The prepared membranes were first compacted by filtering DI water at a pressure of 1.5 bar for 1 h. PWP was calculated using **Eq. (S2):**

$$\mathrm{PWP} = \frac{V}{A\,\Delta t\,\Delta P} \qquad (S2)$$

where V is the permeate volume of water (L), A is the effective area of the membrane (m^2), Δt is the filtration time (h), and ΔP is the applied pressure (bar).

CN^- removal of the prepared membranes was assessed by filtering a solution containing NaCN (50 ppm) dissolved in DI water. The removal (*R; %*) of the fabricated membranes was calculated using **Eq. (S3):**

$$R(\%) = \left(1 - \frac{C_\mathrm{p}}{C_\mathrm{b}}\right) * 100 \qquad (S3)$$

where C_b and C_p are the concentrations of NaCN in the feed and permeate, respectively.

Fluorescence response of Cu-TCPP MONs towards filtered water from Cu-TCPP/CA membranes

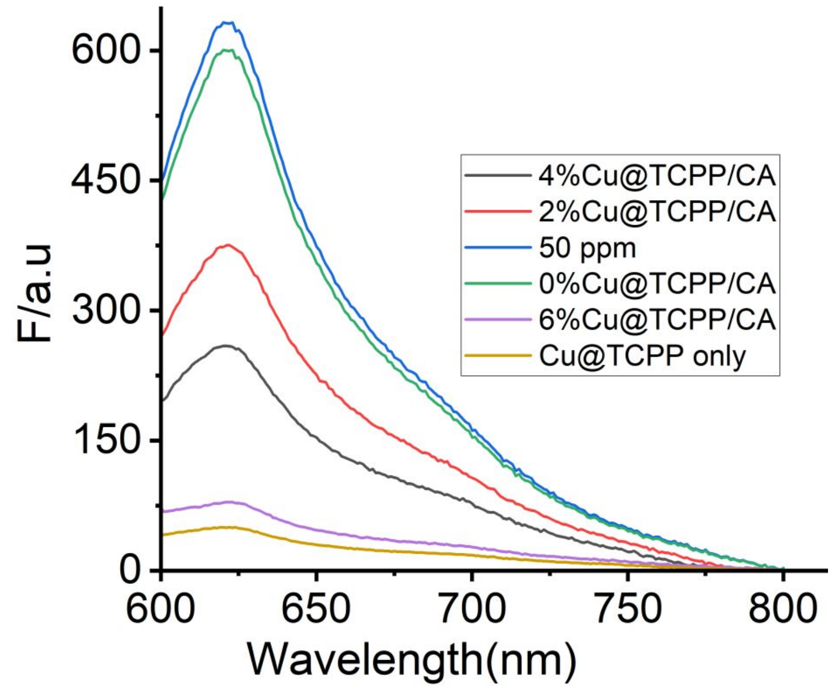

Figure S11. Fluorescence response of Cu-TCPP MONs towards filtered water from Cu-TCPP/CA membranes. In this experiment, 50 ppm NaCN spiked to water passed through the Cu-TCPP/CA membranes. Fluorescence intensity was reduced in water samples collected from membranes with more incorporated Cu-TCPP MONs.

Singlet oxygen generation ability of Cu-TCPP MONs

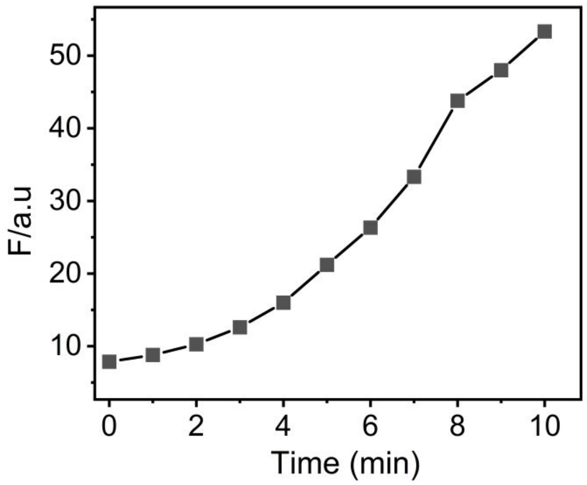

Figure S12. Light-induced ROS generation from Cu-TCPP MONs at equal concentrations using SOSG as a ROS probe. Emission was monitored at 525 nm by using an excitation wavelength of 505 nm under UV light (365 nm) irradiation source.

PXRD and solid-state UV-Vis spectroscopy of 6% Cu-TCPP/CA before and after CN^- loading

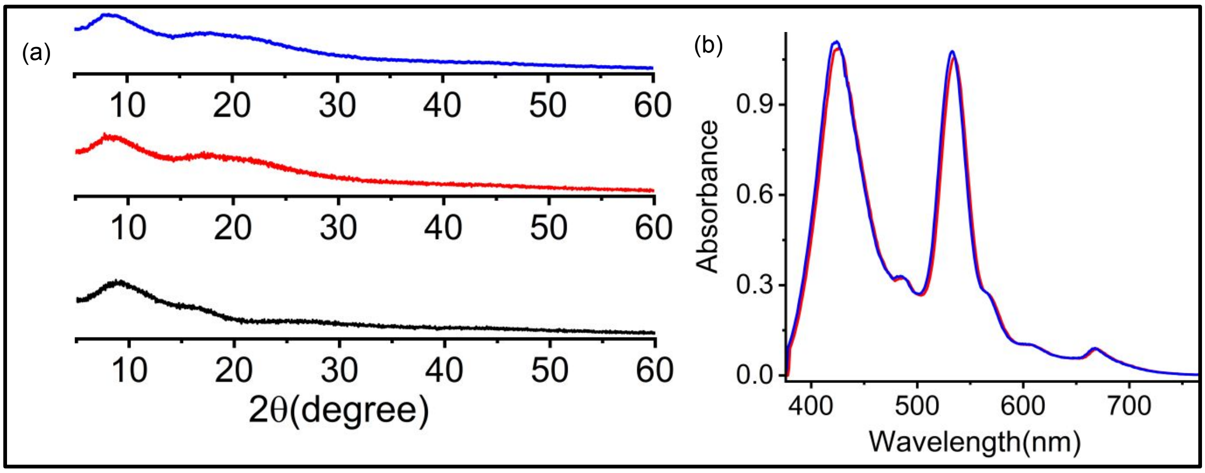

Figure S13: (a) PXRD of 0%Cu-TCPP/CA (black line); 6%Cu-TCPP/CA before (red line) after CN^- loading (blue line); (b) Solid state UV-Vis spectra of 6%Cu-TCPP/CA before (red line) after CN^- loading (blue line).

EDAX analysis of 6% Cu-TCPP/CA before and after CN^-loading

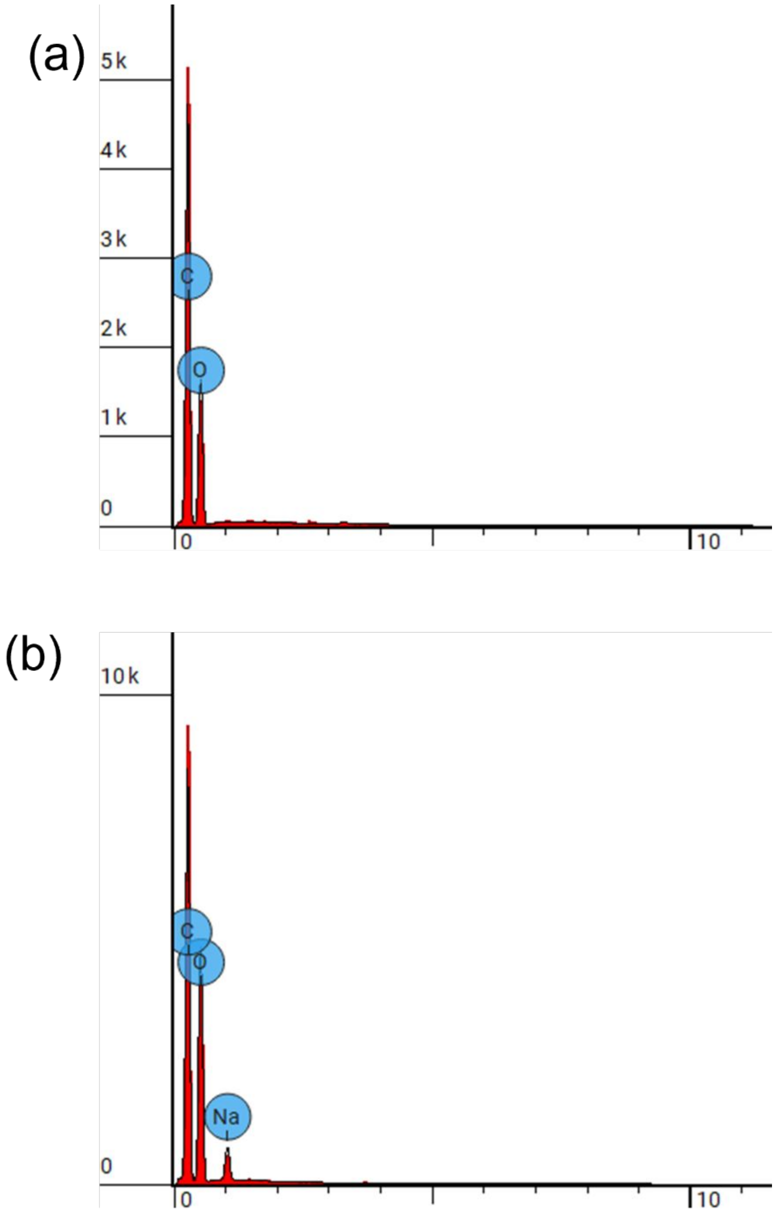

Figure S14: EDAX analysis of 6% Cu-TCPP/CA (a) before and (b) after CN^-loading by using Phenom SEM desktop instrument.

Recyclability

The reusability of Cu-TCPP/CA composite membrane were evaluated by examining the flux recovery ratio (FRR; %) over the course of 3 cycles, with each cycle spanning a duration of 2 h: a 1-h filtration of DI water, followed by a 1-h filtration of the NaCN solution (50 ppm) dissolved in DI water. After each cycling test, the membranes were washed with DI water for 5 min. The FRR was calculated using Eq.[1]

$$FRR\ (\%) = {}^{J_v}\!/_{J_w} \qquad (S4)$$

where, J_w and J_v are DI water flux in first cycle and DI water flux after the completion of the subsequent cycle and washing the membrane, respectively.

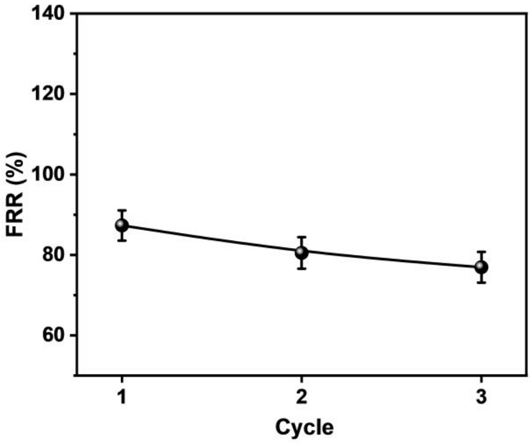

Figure S15: Flux recovery ratio (FRR) of 6%Cu@TCPP/CA membrane after each cleaning cycle.

Pore Size calculation:

Mean pore size (r_m) of membrane without metal-organic nanosheets (0%Cu-TCPP/CA) were calculated using the Guerout-Elford-Ferry method reported in the literature by applying equation (S5).[1]

$$Mean\ pore\ size = \sqrt{\frac{(2.9-1.75\varepsilon)\times 8\eta lQ}{\varepsilon A\Delta P}} \qquad S5$$

where η is pure water viscosity, Q is permeation volume of DI water ($m^3\ s^{-1}$), A is the effective membrane area and ΔP is the applied pressure for permeation.

Reference:

1. Kumar, M.; Sreedhar, N.; Thomas, N.; Mavukkandy, M.; Ismail, R. A.; Aminabhavi, T. M.; Arafat, H. A., Polydopamine-coated graphene oxide nanosheets embedded in sulfonated poly(ether sulfone) hybrid UF membranes with superior antifouling properties for water treatment. *Chemical Engineering Journal* **2022,** *433*.
